# Camelid Single-Domain Antibodies As an Alternative to Overcome Challenges Related to the Prevention, Detection, and Control of Neglected Tropical Diseases

**DOI:** 10.3389/fimmu.2017.00653

**Published:** 2017-06-09

**Authors:** Carla F. C. Fernandes, Soraya dos S. Pereira, Marcos B. Luiz, Juliana P. Zuliani, Gilvan P. Furtado, Rodrigo G. Stabeli

**Affiliations:** ^1^Fundação Oswaldo Cruz, Fiocruz Rondônia, Porto Velho, Rondônia, Brazil; ^2^Departamento de Medicina da Universidade Federal de Rondônia, UNIR, Porto Velho, Rondônia, Brazil; ^3^Fundação Oswaldo Cruz, Fiocruz Ceará, Fortaleza, Ceará, Brazil; ^4^Plataforma Bi-Institucional de Medicina Translacional (Fiocruz-USP), Ribeirão Preto, São Paulo, Brazil

**Keywords:** VHH, single-domain antibody, biotechnology, neglected tropical diseases, camelid antibody

## Abstract

Due mainly to properties such as high affinity and antigen specificity, antibodies have become important tools for biomedical research, diagnosis, and treatment of several human diseases. When the objective is to administer them for therapy, strategies are used to reduce the heterologous protein immunogenicity and to improve pharmacokinetic and pharmacodynamic characteristics. Size minimization contributes to ameliorate these characteristics, while preserving the antigen–antibody interaction site. Since the discovery that camelids produce functional antibodies devoid of light chains, studies have proposed the use of single domains for biosensors, monitoring and treatment of tumors, therapies for inflammatory and neurodegenerative diseases, drug delivery, or passive immunotherapy. Despite an expected increase in antibody and related products in the pharmaceutical market over the next years, few research initiatives are related to the development of alternatives for helping to manage neglected tropical diseases (NTDs). In this review, we summarize developments of camelid single-domain antibodies (VHH) in the field of NTDs. Particular attention is given to VHH-derived products, i.e., VHHs fused to nanoparticles, constructed for the development of rapid diagnostic kits; fused to oligomeric matrix proteins for viral neutralization; and conjugated with proteins for the treatment of human parasites. Moreover, paratransgenesis technology using VHHs is an interesting approach to control parasite development in vectors. With enormous biotechnological versatility, facility and low cost for heterologous production, and greater ability to recognize different epitopes, VHHs have appeared as an opportunity to overcome challenges related to the prevention, detection, and control of human diseases, especially NTDs.

## Monoclonal Antibodies (mAbs): From Biotechnological Assays to Biopharmaceutical Market

The advent of hybridoma technology provided a great development in antibody engineering (1). Using this technology, mAbs with high specificity for proteins, nucleic acids, carbohydrates, and haptens have been produced satisfactorily over time (2). The specificity and affinity of mAbs, added to their homogeneity and unlimited availability, are essential to their applications in the biological sciences (3–5). However, for clinical application, these structures may have some disadvantages, such as low tissue distribution, adverse reactions related to non-human origin proteins (6, 7), and high cost of scale-up production.

Thus, the humanization of heterologous immunoglobulins (Igs), either by fusing the variable regions of mouse antibodies with the constant domains of human Igs or by inserting the murine CDRs into human variable chains, has been an alternative for increasing the effectiveness and safety of immunotherapy. Furthermore, recombinant DNA technology has allowed for the production of different antibody formats for use in biomedical studies, construction of biosensors, and/or therapy (8).

The strategy to minimize antibody size preserves antigen–antibody binding sites generating monovalent [fragment antigen binding (Fab)] fragments from IgGs, single-chain variable fragments (scFv), as well as single-domain antibodies (9). This approach aims to improve bioavailability and reduce immunogenicity, when the objective is to use them for pharmacological treatment. When these fragments are designed for radioimmunotherapy or *in vivo* imaging, the lack of a constant region [fragment crystallizable (Fc)] allows for better renal clearance (10). For diagnostic methods, like immunohistochemistry, the lack of Fc ensures, in addition to better tissue distribution, reduced non-specific binding.

It is important to mention that antibody fragments normally lack glycosylation, allowing for their production in prokaryotic expression systems, saving time and money (9). However, fragments lacking an Fc domain possess a shorter half-life than intact IgGs and are unable to elicit Fc-mediated cytotoxic processes (11), which require optimizations for antibody fragment products.

Since the introduction of the first therapeutic mAb (Orthoclone OKT3) in the biopharmaceutical market in 1986, several monoclonal antibody therapeutics have been approved for the treatment of diseases (2). After 30 years, the global sales revenue of biopharmaceutical products is nearly $150 billion. From these, about 50% are related to the sales of therapeutic mAbs or related products, including antibody fragments, antibody-drug conjugates, and Fc-fusion proteins (12).

Currently, about 50 monoclonal antibody products have been approved by the Food and Drug Administration, in the US, or European Medicines Agency in Europe, for the treatment of several diseases (12). Among the products, most of them were developed to treat chronic inflammatory diseases and cancer, but there are also products in the market for neurodegenerative disorders and for infectious diseases (13).

Advances in the understanding of disease physiopathology at a molecular level, together with recombinant DNA technology, and the intrinsic characteristics of antibodies, point to significant growth in this sector of the pharmaceutical market over the next few years (14). A rate of four to six new products per year is expected in the market, reaching about 70 monoclonal products in 2020, with worldwide sales of approximately $ 125 billion (12, 15).

## Camelid Heavy-Chain Antibodies (HCAbs) and VHH Domain

The discovery that the Camelidae family produces a significant proportion of functional antibodies lacking L chains, along with molecular technologies, consolidated at the beginning of twenty-first century brought new perspectives for the antibody-bioengineering field (16, 17).

Referred to as camelid HCAbs (IgG_2_ and IgG_3_), these molecules have approximately 90 kDa, and the antigen recognition site is formed by a single domain, termed VHH (variable domain of camelid heavy-chain-only antibody) or nanobody (16, 18). The percentage of HCAbs in the total IgG of these animals represents 10–80%, indicating the importance of these antibodies in the immune protection of camelids (18, 19).

**Figure 1:**
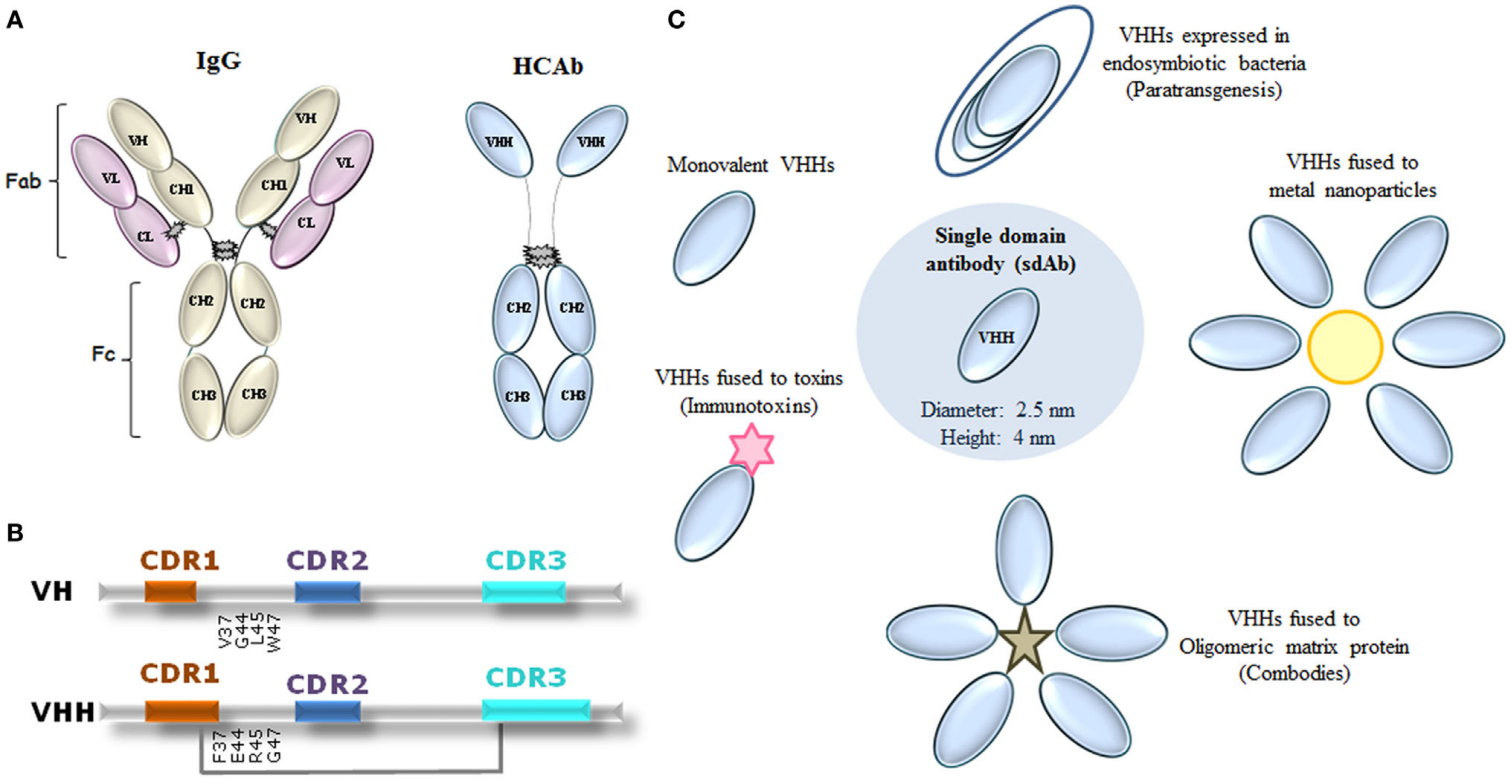
Representation of immunoglobulin (Ig) G molecules and uses of VHHs in neglected tropical diseases (NTDs). **(A)** Conventional IgG showing heavy and light domains that perform fragment antigen binding (Fab) and fragment crystallizable (Fc) regions, and camelid heavy chain [heavy-chain antibody (HCAb)] IgG. **(B)** Comparison between VH and VHH domains on the amino acid sequence level. CDR1 and CDR3 of VHHs are often larger than the respective VH regions. Amino acid substitutions in framework 2 are given, as well as an eventual extra disulfide bond between CDR1 and CDR3 (gray line). CDR1 indicated as red; CDR2 as blue, and CDR3 as light blue. Adapted from Ref. (24, 27–29). **(C)** Proposed uses of VHHs in NTDs. VHHs have been proposed in the following formats: monovalent, fused to proteins (immunotoxins), fused to oligomeric matrix proteins (combodies), conjugated to metal nanoparticles, and expressed by endosymbiotic bacteria in NTD vectors for a paratransgenic approach.

VHHs present a similar organization to that of VHs from conventional IgGs (1, 20). Both are composed of three variable CDRs interspersed with four conserved FRs, but with notable differences. To improve the molecular stability of VHHs, hydrophobic amino acid residues present in conventional VHs (FR2 regions) were substituted for smaller or more hydrophilic amino acids in VHHs (21–24). Besides that, CDRs 1 and 3 are usually larger, providing a compensatory antigen interaction surface in the absence of the VL domain (18, 25). This difference is observed mainly in the CDR3, which possesses an average of 18 amino acid residues, in contrast with about 14 residues in the VH orthologous region. Additional disulfide bonds formed frequently between CDR3 and CDR1 or FR2 restricts the flexibility of the prominent loop, ensuring greater stability for these molecules (26) (Figures 1A,B).

When compared to scFv or disulfide-stabilized Fv antibody fragment, VHHs present higher thermal stability, maintaining binding activity between 80 and 100% (30). Paving the biotechnological way, at high temperatures, conventional antibody fragments expose hydrophobic interfaces between VH and VL, inducing aggregation and precipitation. VHH possess a great thermal stability, and it may be related to the replacement of the hydrophobic amino acids (31).

With one-tenth the size of IgGs, nanometer dimensions, and extended CDRs, VHHs are capable of penetrating dense tissues and interacting with weakly antigenic epitopes for human or murine antibodies. A similarity greater than 80% with the FRs of human VH regions and high renal clearance justify their low immunogenicity (20, 32). Additionally, their high solubility, affinity, and specificity for molecular targets, stability, flexibility related to the construction of different formats (monomers, dimers—mono- or bispecifics, fused to drugs, etc.), multiple routes of administration, humanized construction possibilities, and low cost of production make VHHs important biotechnological tools.

Combining the advantages of mAbs with properties of small molecules, the possibilities of VHH applications in health are vast. Beyond the development of drugs for inflammatory and neurodegenerative diseases, along with antitumor and viral neutralizing agents, VHHs are useful for drug delivery and are interesting agents in diagnosing diseases (33–37). Furthermore, these fragments could be used as intrabody, directed to specific intracellular target proteins, aiming, for example, to inhibit viral replication (38, 39). In the field of serum therapy, VHHs have emerged as tools for antivenom development (40–43).

Although no therapeutic agent is currently available, several VHH-based products are under development. The company Ablynx, in collaboration with multiple pharmaceutical industries, has 40 product candidates for the treatment of cancer, inflammatory, and neurodegenerative diseases. Five products are in clinical development, and the first therapeutic agent based on VHH, anti-vWF, is expected to be sold in 2018 (44). Despite the expected increase in antibody development over the next years, many diseases remain neglected because drugs or diagnostic tools would not generate monetary profits for the big pharmaceutical companies (45, 46). Recently, public–private partnerships have emerged to develop medicines and health technologies to circumvent the challenges faced in NTDs (47).

## Diagnostic and Treatment Challenges in NTDs

Neglected tropical diseases are a group of infectious diseases, caused by parasites, viruses, and bacteria, prevalent in 149 countries affecting more than one billion people (48). Most of these people live in extreme poverty without adequate sanitation and in close contact with infectious vectors and domestic animals and livestock. Causing relatively low mortality but high morbidity, NTDs generate serious consequences for individuals or entire communities in terms of lifestyle quality, loss of productivity and poverty aggravation, and cost billions of dollars every year to developing economies (49).

Today, 19 diseases are classified as NTDs, including Buruli ulcer, Chagas disease (CD), dengue, chikungunya, dracunculiasis, echinococcosis, foodborne trematodiases, human African trypanosomiasis (HAT), leishmaniasis, leprosy, lymphatic filariasis, onchocerciasis, rabies, schistosomiasis, soli-transmitted helminthiases, taeniasis/cysticercosis, trachoma, yaws, and mycetoma. Moreover, WHO recognizes that there are still many tropical and poverty-related diseases or conditions that remain neglected and also require adequate prevention and control approaches (48).

These diseases can be preventable and treatable by combining public political strategies. Besides provision of safe water, sanitation and hygiene (WASH), personal protection measures, and rigorous implementation of vector control are required in reducing disease transmission (47). Integrative actions between NTD and WASH programs, in addition to the mass drug administration (MDA), create conditions for more effective control of many NTDs (50).

However, for most NTDs, the diagnostic methods are limited, the MDA treatment regimen presents limitations, and the medications used are archaic presenting high toxicity or inadequate efficacy (51). Thus, safe and effective drugs, vaccines, and diagnostic devices remain important challenges to prevent or control the course of NTDs. Given the necessity to develop fast and effective diagnostic methods, as well as innovative approaches to treat NTDs, and the increasing use of antibody-based products in health, research groups have invested in the development of these alternatives to enhance the fight against NTDs (52–54). Although promising, the high cost of mAb production compromises its large-scale manufacture, especially for NTDs. Moreover, VHHs’ superior ability to recognize and neutralize antigens as compared to VHs of Fabs or scFvs, in addition to their lower production cost, instigates the development of VHH-based products for these diseases.

## Versatile VHH Approaches for NTDs

Like lego bricks, VHH technology has used the modular concept for several antibody-based applications. Among the proposed uses of VHHs for NTDs are monovalent or conjugated VHH structures for recognizing or inhibiting selected targets (Table 1). As conjugated structures, VHHs could be fused to metal nanoparticles, especially gold, for construction of fast immunochromatographic tests; fused to oligomeric matrix proteins (Combodies), to amplify the affinity and ability in neutralizing viruses; conjugated with proteins able to lyse infectious agents, so-called immunotoxins; and expressed by endosymbiotic bacteria in NTD vectors, as potential *in vivo* drug delivery systems, a paratransgenic approach (Figure 1C).

**Table 1:** Overview of published camelid single-domain antibodies developed against neglected tropical disease (NTD).

NTD	Target	Camelid	Producing host	Proposed use	Reference
Chagas disease	Recombinant trans-sialidase (TcTS)	Llama	*E. coli*	Alternative chemotherapy	(55)
Dengue	NS1 protein (dengue virus—type 2)	Llama	*E. coli*	Point-of-care devices	(56)
Human African trypanosomiasis (sleeping sickness)	*Trypanosoma brucei brucei* specific variant surface glycoprotein (VSG)	Dromedary	*E. coli*	Alternative treatment	(57, 58)
	Anti-VSG VHH conjugated with a truncated apolipoprotein L-I	Dromedary	*E. coli*	Alternative treatment	(59)
	*T. brucei brucei* specific VSG	Dromedary	*Sodalis glossinidius*	Alternative treatment—paratransgenesis	(60, 61)
	*T. brucei* KREPA6 multi-functional editosome protein	Llama	*E. coli*	Structural protein studies	(62)
	Paraflagellar rod protein	Alpaca	*E. coli*	Diagnostic tool—one-step direct immunofluorescence	(63)
	Tsal protein	Alpaca	*E. coli*	Diagnostic tool—competitive immunoassay	(64)
Leishmaniasis	Endonuclease G	Llama	*E. coli*	Research tool	Creative Biolabs NAB-817-sdAb^a^
Rabies	Glycoprotein G	Llama	*E. coli/Pichia pastoris*	Postexposure prophylaxis	(35, 65–67)
Schistosomiasis	*S. mansoni* Cathepsin B and excretory/secretory antigen	Camel	*E. coli*	Diagnostic and therapeutic applications	(68)
Cysticercosis	*Taenia solium* Ts14 glycoprotein	Dromedary	*E. coli*	Specific diagnostic assay	(69)

*^a^Data found on the commercial website of the company Biolabs (http://www.creativebiolabs.net/Anti-Leishmania-EndoG-VHH-Single-Domain-Antibody-54.htm)*.

### VHHs As a Tool against CD

*Trypanosoma cruzi* is the etiological agent of CD, an anthropozoonosis endemic to the American continent (70, 71), that affects about 10–12 million people worldwide (72). One of the mechanisms used by *T. cruzi* to survive in the mammalian host is related to the presence of highly diverse glycosylated mucins in parasite membrane (73, 74). Membrane anchored trans-sialidase (TcTS) participates in the addition of sialic acid in mucin coat, and so it is an important protein involved in the pathogenesis of *T. cruzi* (55, 75–77). TcTS activity was neutralized by antibodies found in patients with chronic CD, as well as in animals infected with *T. cruzi* (78–80). Passive transfer of anti-TcTS mAbs to infected animals prevents thrombocytopenia induced by the enzyme (81). Given the VHHs are often potent enzyme inhibitors, Ratier and colleagues (55) produced anti-TcTS VHHs (55). Despite inhibiting recombinant TcTS, the selected VHHs failed to neutralize purified TcTS from *T. cruzi* parasites. These results point to the relevance of diversity among members of the TcTS family (55, 82), which may be required for *T. cruzi*’s strategy to evade the host immune system.

### VHHs As a Tool against Dengue

Belonging to the *Flavivirus* genus, the dengue virus (DENV) can cause visceral and central nervous system disease in humans. Each year, about 390 million people are infected with DENV and more than 3.6 billion people live in at-risk areas (83, 84). Currently, four DENV serotypes have been identified (83). Besides humans, the main vectors of the virus in nature are mosquitoes from the genus *Aedes* (73). These vectors contribute not only to Dengue outbreak but also to the spread of other mosquito-borne diseases, like Zika and chikungunya (85, 86). Vaccines for these diseases are at different stages of development, no specific treatment is available, and diagnostic methods are limited. Among the initiatives for the development of rapid diagnostic kits, Fatima and coworkers (56) produced VHHs and mAbs against DENV (type 2) NS1 non-structural protein aiming to construct two diagnostic kits based on an immunochromatographic assay (56). Comparing both devices, the VHH-based kit demonstrated better sensitivity and specificity against the antigen than the mAb-immobilized kit. Although the two antibodies recognize the same protein epitope, better results related to VHH devices might be due to VHHs’ longer CDR3 and their capability to bind to the cleft of the targeted antigen. This study demonstrated the viability of VHH-based point-of-care tests for the detection of DENV infection.

### VHHs As a Tool against HAT (Sleeping Sickness)

About 300,000 human beings suffer from HAT, more than 60 million people live in areas with risk of infection (87). While *Trypanosoma brucei gambiense* causes the chronic form of the disease in western and central Africa, *T. brucei rhodesiense* is the etiological agent of an acute disease in eastern and southern Africa (88). Although the parasite and its vectors (tsetse flies, *Glossina* spp.) were identified more than a century ago, control of the disease remains elusive. With few drugs available, often causing toxicity, along with increasing drug resistance, the search for alternatives to treat the disease has become urgent (88, 89). Antigen variation is a defense mechanism adopted by trypanosomes to escape from the host–immune system. These parasites express numerous copies of the variant surface glycoprotein (VSG) in their membrane (88). Taking advantage of VHH properties, Stijlemans et al. (57) showed that anti-VSG VHHs were able to penetrate the VSG coat to target their epitope (57). Furthermore, Baral et al. (59) fused VHHs with truncated human trypanolytic factor [apolipoprotein L-I (apoL-I)] and demonstrated that the immunotoxins were capable of trypanolysis (59). Treatment with apoL-I–anti-VSG VHH conjugates resulted in curative and alleviating effects on acute and chronic infections in mice with both resistant and sensitive trypanosome strains. Caljon et al. (58) indicated that anti-VSG VHHs could penetrate the brain–blood barrier, especially in pathological conditions (58). Another strategy suggested to handle HAT is related to the control of trypanosome development in tsetse flies. Thus, De Vooght et al. (60), exploring paratransgenesis, expressed anti-VSG VHHs in the bacteria *Sodalis glossinidius*, an endosymbiotic microorganism of the fly (60). Subsequently, the group demonstrated that recombinant *S. glossinidius* can release anti-VSG VHHs in different tissues of *Glossina morsitans morsitans* (61). Beyond effective treatment, HAT requires efficient diagnostic devices. So, anti-paraflagellar rod protein (PRP) VHHs of trypanosomes or anti-Tsal protein, a biomarker present in tsetse fly saliva, seem to be alternatives for diagnosing the disease. While anti-PRP VHHs are suggested for the development of IFA assays, a T-sal VHH-based competitive immunoassay could be used to identify tsetse fly exposure (63, 64). Furthermore, anti-KREPA6 multifunctional protein of editosome VHHs of *T. brucei* has been proposed for use in structural protein studies (62).

### VHHs As a Tool against Rabies

Rabies virus (RABV) causes approximately 59,000 human deaths per year (90). Shortly after exposure, patients should receive anti-rabies prophylaxis, which consists of passive immunization, with rabies-specific Igs, and a vaccine (65, 91). Issues related to the possibility of infectious agent transmission, high cost, and limited production, as well as the need for special storage conditions, instigate the development of new strategies for the disease. In 2015, the WHO and other groups announced a goal to eliminate rabies deaths worldwide by 2030. They call for cheaper and faster treatment for people and improved vaccinations in domestic dogs (90).

Since the outer envelope RABV glycoprotein was identified as the most significant surface antigen for generating virus-neutralizing antibodies, researchers have selected this target as a strategy to build broadened neutralizing antiviral molecules. In 2011, Hultberg and coworkers developed the first anti-RABV VHHs (35). VHH constructs improved the cross neutralization against viral strains, as well the viral neutralization potencies up to 1,500-fold, which was similar to or better than the best performing mAbs. Boruah and coworkers (66) fused anti-RABV VHHs with a peptide derived from the human cartilage oligomeric matrix protein (COMP48) to construct pentavalent multimers, called combodies (66). These combodies conferred protection for mice infected with lethal doses of RABV. Posteriorly, Terryn et al. (67) demonstrated the protective effect of VHH-based constructs against rabies induced in mice (67). Besides prolonging animal survival, anti-rabies VHHs were able to rescue mice from the disease. Construction of bivalent or biparatopic VHHs resulted in increased neutralizing potency, reaching a picomolar range. Recently, the same group showed that PEP using anti-RABV VHHs associated with vaccine administration improves protection of mice from lethal rabies infection. It is important to note that the vaccine alone, as well as the association of anti-RABV Igs with the vaccine, was unable to rescue mice from the lethal disease (65).

### VHHs As a Tool against Schistosomiasis

Schistosomiasis, caused by a parasite of the genus *Schistosoma*, affects almost 240 million people worldwide. Infection is acquired when parasitic larvae (cercariae) penetrate the skin of people in contact with infested water. Based on the need to develop new approaches to schistosomiasis diagnosis and treatment, Sallam (68) developed an anti-schistosoma VHH–nanoparticle conjugate, using *S. mansoni* Cathepsin B and execratory secretory proteins, able of recognizing antigens from this parasite (68).

### VHHs As a Tool against Taeniasis/Cysticercosis

Cysticercosis is a parasitic infection caused when humans ingest eggs of the pork tapeworm *Taenia solium*, and the larval form moves through the body and forms cysts in tissues, including the brain. With an objective of creating a more efficient diagnostic method, Deckers et al. (69) developed a specific VHH capable of discriminating the *T. solium* parasite from other *Taenia* species (69). The VHH recognizes a 14 kDa glycoprotein (Ts14) with subnanomolar affinity, using an antigen extract in the immunization of the animal. Purified polypeptides for immunization and panning could obtain VHHs with higher affinities, increasing the diagnostic/therapeutic use of anti-taenia VHHs, mainly in countries with critical sanitation problems.

## Conclusion and Perspectives

Prophylactic and therapeutic aspects of various NTDs need more effective, cheaper, and faster approaches since currently there are limited methods for diagnosis, in some cases no vaccines, and the usual medications can cause systemic toxicity, besides constantly increasing drug resistance. Camelid VHHs conserve characteristics such as affinity and specificity of conventional IgGs, possess superior thermal stability, low immunogenicity, interesting pharmacokinetic and pharmacodynamic properties, biotechnological versatility, and can be cost-effectively produced in microorganisms.

All these attributes make VHHs valuable candidates for the development of alternative diagnostic tools, such as point-of-care devices, or safe drugs for a diverse number of diseases. Furthermore, bioinformatic algorithms, strategies of protein-driven evolution, and synthetic biology favor obtaining antibody molecules with improved properties. While researchers, mainly from Europe, are focused on the development of VHH-based products for inflammatory, cancer, and neurodegenerative disorders, the Oswaldo Cruz Foundation (Fiocruz) in Brazil established a VHH-development platform to produce these products aiming at the diagnosis or treatment of tropical diseases, mainly emerging arboviruses, as Zika, chikungunya, and yellow fever. To transform research results into products with high aggregate, social value is the big challenge in the area, since NTDs affect populations of the world’s smallest economies.

## Author Contributions

CF made substantial contributions to the conception, design, and drafting of the manuscript; SP contributed to the preparation and revision of the manuscript; ML participated in drafting the article; JZ contributed to the writing and revision of the manuscript; GF made substantial contributions to design, acquisition of data, and revision of the manuscript; and RS participated in drafting the article and reviewed it critically for intellectual content.

## Conflict of Interest Statement

The authors declare that the research was conducted in the absence of any commercial or financial relationships that could be construed as a potential conflict of interest. The reviewer, JA, and handling editor declared their shared affiliation, and the handling editor states that the process nevertheless met the standards of a fair and objective review.
